# Evaluating Hyperspectral Vegetation Indices for Leaf Area Index Estimation of *Oryza sativa* L. at Diverse Phenological Stages

**DOI:** 10.3389/fpls.2017.00820

**Published:** 2017-05-22

**Authors:** Mairaj Din, Wen Zheng, Muhammad Rashid, Shanqin Wang, Zhihua Shi

**Affiliations:** ^1^College of Resources and Environmental Sciences, Huazhong Agricultural UniversityWuhan, China; ^2^Key Laboratory of Arable Land Conservation (Middle and Lower Reaches of Yangtze River), Ministry of Agriculture, Huazhong Agricultural UniversityWuhan, China; ^3^Plant Breeding and Genetics, Nuclear Institute for Agriculture and BiologyFaisalabad, Pakistan

**Keywords:** rice, LAI, phenology, N-nutrition, hyperspectral reflectance

## Abstract

Hyperspectral reflectance derived vegetation indices (VIs) are used for non-destructive leaf area index (LAI) monitoring for precise and efficient N nutrition management. This study tested the hypothesis that there is potential for using various hyperspectral VIs for estimating LAI at different growth stages of rice under varying N rates. Hyperspectral reflectance and crop canopy LAI measurements were carried out over 2 years (2015 and 2016) in Meichuan, Hubei, China. Different N fertilization, 0, 45, 82, 127, 165, 210, 247, and 292 kg ha^-1^, were applied to generate various scales of VIs and LAI values. Regression models were used to perform quantitative analyses between spectral VIs and LAI measured under different phenological stages. In addition, the coefficient of determination and RMSE were employed to evaluate these models. Among the nine VIs, the ratio vegetation index, normalized difference vegetation index (NDVI), modified soil-adjusted vegetation index (MSAVI), modified triangular vegetation index (MTVI2) and exhibited strong and significant relationships with the LAI estimation at different phenological stages. The enhanced vegetation index performed moderately. However, the green normalized vegetation index and blue normalized vegetation index confirmed that there is potential for crop LAI estimation at early phenological stages; the soil-adjusted vegetation index and optimized soil-adjusted vegetation index were more related to the soil optical properties, which were predicted to be the least accurate for LAI estimation. The noise equivalent accounted for the sensitivity of the VIs and MSAVI, MTVI2, and NDVI for the LAI estimation at phenological stages. The results note that LAI at different crop phenological stages has a significant influence on the potential of hyperspectral derived VIs under different N management practices.

## Introduction

The application of remote sensing technology inprecision agriculture management has become increasingly prevalent among farmers due to its ability to optimize crop status by facilitating sound crop monitoring (Pei et al., 2014). In recent decades, the development of crop canopy sensors has enabled precision agriculture to be used for non-destructive estimation of crop biophysical attributes in fields or even at the regional scale (Diacono et al., 2013). Remote sensing can generate useful spectral reflectance data that provide rapid means for monitoring growth status through various biophysical, physiological, or biochemical crop parameters. For crop dynamic monitoring, timely observation of the plant biophysical properties and ecophysiological status, e.g., leaf area, light use efficiency, chlorophyll, and nitrogen contents, has become crucially important to enhance nutrition and improve yield for universal food sanctuary and sustainable development (Zhao et al., 2013).

Rice (*Oryza sativa* L.) is a staple food crop for global food security, providing food for over 3 billion people and more than 20% of their daily calorie intake (Luck et al., 2011; Seck et al., 2012). During rice canopy development, a photosynthetic photon flux density (PPFD) gradient can provide accurate description of the physiological relationship between nitrogen content and leaf area distribution vertically in canopy (Yang et al., 2014). But the construction of PPFD is time-costing and difficult to measure parameters. The leaf area index (LAI), the one-sided green leaf area per unit ground surface area, is a key biophysical variable that is directly involved with canopy functioning processes, such as photosynthesis and respiration (Casa et al., 2012). It is a necessary parameter used by crop physiologists to remotely estimate canopy cover, crop growth and yield. Moreover, it is functionally linked to the canopy spectral reflectance (Jin et al., 2013).

A healthy plant canopy visually appears green because the leaf pigments strongly absorb blue and red light and reflect green light, and the reflectance curve in the red and blue region shows a valley. Thus, increased reflectance at the near infrared region was more related to the vegetation cover, biomass, leaf internal cell structure, water content of the leaf, and LAI, while the boundary of the red region has strong absorption due to the leaf chlorophyll, N concentration, and reflection due to mesophyll cells in growing plants (Datt, 1998). A number of approaches have been used to address the relationship between biophysical parameters and canopy reflectance (Lemaire et al., 2008; Gitelson et al., 2014; Padilla et al., 2014), but recently two main strategies have use to estimate the LAI estimation using spectral data: (1) the empirical relationship between spectral vegetation indices (VIs) and biophysical variables and (2) inversion of canopy radiative transfer models, such as PROSAIL model (Jacquemoud et al., 2009). The latter strategy uses complicated models because they do not account for as much of the optimized variability caused by the large spatial coverage of biophysical variables (Ryu et al., 2009). However, more effective ways to predict the LAI from spectral data are based on the first strategy to explore the empirical relationship between VIs verses LAI (Xie et al., 2014). These methods are computationally undemanding and sequentially simple to employ while capturing broad array of variation in crop canopy features, and widely used to estimate vegetation biophysical variables, including the LAI (Le Maire et al., 2008). However, broad-band VIs (band width > 50 nm) are often affected by high soil and water background reflectance when the vegetation canopy is sparse. Furthermore, the integrating processing of spectra to derive broad-band data result in loss of detail of vegetative spectral response (Broge and Leblanc, 2000). So, the narrow-band indices (band width < 10 nm) were considered as promising ways for improve accuracy of estimation of canopy parameters (Li et al., 2012).

Hyperspectral reflectance provides measurements over numerous narrow wavelength bands (<10 nm) that contain additional bands within the visible, near infrared, and short wave infrared region of the spectrum (400–2500 nm). Moreover, hyperspectral reflectance data make it possible to collect more than 100 bands at high resolution (Sahoo et al., 2015). Hyperspectral reflectance has thus been used to identify the regions of the spectrum that are sensitive to the LAI and least affected by exogenous factors (Ryu et al., 2011). Thus, the selection of important wave bands in hyperspectral data for the constriction of more specific VIs is the key to maximizing the efficacy of LAI estimation (Ryu et al., 2009). Moreover, combinations of these wave bands provide optimal information about LAI characterization with the phenological stages of the crop under varying environmental conditions and cultural practices (Nakanishi et al., 2012).

Agronomically, rice has three growth stages: vegetative, reproductive pre-heading, and reproductive post-heading. The vegetative stage is phase from germination to panicle initiation, the pre-heading phase is from panicle initiation to heading, and the post-heading phase is from heading to maturity. Canopy spectral reflectance at the phenological stage of tillering and elongation under different canopy sensors shows that some VIs are significantly associated with the rice phenological stages (Bajwa et al., 2010). The relationship between VIs and the LAI at these phenological stages greatly contributes to estimating biomass accumulation and evaluating the N status of rice plants (Motohka et al., 2010). Consequently, it is indispensable for investigating the relationship between VIs and phenological stages under diverse nitrogen dynamics (Sadras and Lemaire, 2014). In our study, the main objective was to evaluate the potential of various VIs in relation to the LAI at phenological stages using data collected over 2 years. This study also focuses on the variation of LAI over phenological periods, which is important for N fertilization management. The expected results would help to provide a technical approach for non-destructive LAI monitoring and to develop a simple, rapid, and cost-effective N management strategy at different phenological stages of rice.

## Materials and Methods

### Experimental Site

**FIGURE 1:**
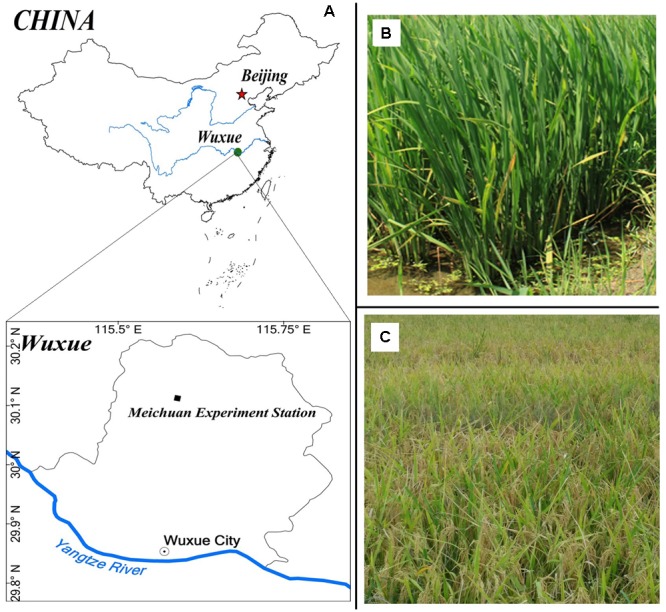
**Photographs of mid season rice in 2015 and 2016 (A)**. Meichuan Experimental Station and field view **(B)**. Before heading **(C)** at maturity.

Two-year field experiments were conducted in two different fields at the experimental station of Huazhong Agricultural University in Meichuan town, located (30° 06′ N, 115° 35′ E) in Wuxue, Hubei, China, from May to October in 2015 and 2016 (**Figure 1**). The area has a subtropical moist monsoon climate with a mean annual temperature and precipitation of 17.7C and 1903 mm, respectively, with summer being the driest period and autumn being the wettest period. The pH of the soil is 5.14. The soil contained 26.50 g/kg organic matter, 1.57 g/kg total N, 11.6 mg/kg available phosphate (P_2_O_5_), and 137.5 mg/kg available potassium (K_2_O) at the layer of 0–20 cm.

The experiment was conducted as a randomized complete plot design (RCBD) with eight levels of nitrogen in three blocks (replicates). Treatments used various N fertilizer rates, 0, 45, 83, 128, 165, 210, 248, and 293 kg N ha^-1^ urea (N concentrations 46%), for each plot of, which was 20 m^2^ N application was distributed as 55% for pre-planting, 20% for jointing dressing, and 25% for booting dressing. To prevent water and fertilizer flow between neighboring plots, 40 cm wide ridges covered with plastic film inserted in 30 cm of soil were used to separate each plot. Before transplanting, 90 kg ha^-1^ of P_2_O_5_ from SSP (P_2_O_5_ content 12%) and 180 kg ha^-1^ of K_2_O from potassium (K_2_O content 60%) were applied. Twenty-seven to thirty-day old seedlings of Japonica rice cultivar Shenliang you 5814 were transplanted with a spacing of 0.24 m × 0.30 m to maintain a planting density of approximately 26 hills per m^2^. Following the local rice production standards, weeds, insects and diseases were strictly controlled throughout the rice growth periods.

### Canopy Spectral Reflectance Measurements

Spectral reflectance measurements were assessed between the hours of 10:00 and 14:00 China Standard (UTC+8) under a clear and cloudless sky during the early (Transplanting) to late (before maturity) growing stages in 2015 and 2016. Canopy spectra were acquired with a portable Field spec (FRTM) spectro-radiometer, an analytical spectral device (ASD, Boulder, CO, United States) that covers the 350–2500 nm spectral range (Pimstein et al., 2011). The radiometer sensor head was positioned 1 m above the canopy, centered over the rice hills, with a nadir field of view of 25°. In 2015 and 2016, three spectral measurements (two at corners and one at the center) were performed within each plot to cover the entire plot. The radiance from a Spectral on reference panel (BaSO_4_) was acquired to derive the reflectance, which was used to calibrate the instrument at 15–30 min intervals prior to each plot reflectance measurement (Mahajan et al., 2014).

In 2015, spectra were collected on five dates between the beginning of tillering (25 DAT), elongation (35 DAT), booting (45 DAT), heading (55 DAT), and 10 DAH (65 DAT) to maturity (July 10 and 28, August 11 and 28, September 11) for all experimental plots.

In 2016, measurements were acquired at the same plane described above at the growth stages, e.g., tillering, elongation, booting, and heading, on July 7 and 23, August 15 and 30 and September 13 for all plots.

Finally, the spectral data were exported to RS2 (ASD, Boulder, CO, United States) software and averaged for each plot. The data were reduced at the edges and for three different spectral portions (e.g., 1341–1439, 1791–1959, and 2401–2500 nm) due to the large noise caused by water absorption in the raw spectrum (Abdel-Rahman et al., 2010).

### Determination of the Field LAI Value

In both years, on the same dates as the spectra acquisitions, the non-destructive LAI was measured using a Plant Canopy Analyzer (LAI-2000, Li-Cor, Inc., Lincolin, NE, United States) over all the plots (Stroppiana et al., 2009). The LAI-2000 is among the most widely used advanced canopy LAI analyzers for many crops, such as cotton, soybean, and maize (Hirooka et al., 2016); in particular, it can be employed to measure leaf growth and perform LAI estimation in different rice cultivars under varying N fertilization regimes.

### Plant Sampling and Measurement

Each plot consisted of two sub-sampling points, and five rice plants from each sub-sampling point were picked without their roots at each phenological stage after acquisition of spectral data. Before each sampling, the tiller number of each hill within the plot was counted (except border rows) for the average tiller number per hill. After removing the panicles (after heading), the fresh plant samples were put into plastic bags and moved to the laboratory. From all of the fresh samples, green leaves were separated from the stems and weighted, and then, all of the samples were placed in a ventilated oven for 30 min at 105°C and dried at 75°C until they reached a constant weight.

The dried samples were milled to pass a 1-mm screen and were then stored in plastic bags for nitrogen analysis. Plant samples of 0.5 g were digested with 3 g of catalyst of 3:1 K_2_SO_4_:CuSO_4_ for at least 6 h at 375°C, along with 10 ml of H_2_SO_4_ and 2 ml of H_2_O_2_. The total N concentration in the leaf tissue was determined by a Flow Injection Analyser (Germany SEAL) three times, and LNC (g^-1^ LDW) was calculated on the basis of the unit dry weight. Then, leaf nitrogen accumulation was computed as the product of LNC (%) and leaf dry weight (LDW, g DW m^-2^).

### Spectral Vegetation Index

**FIGURE 2:**
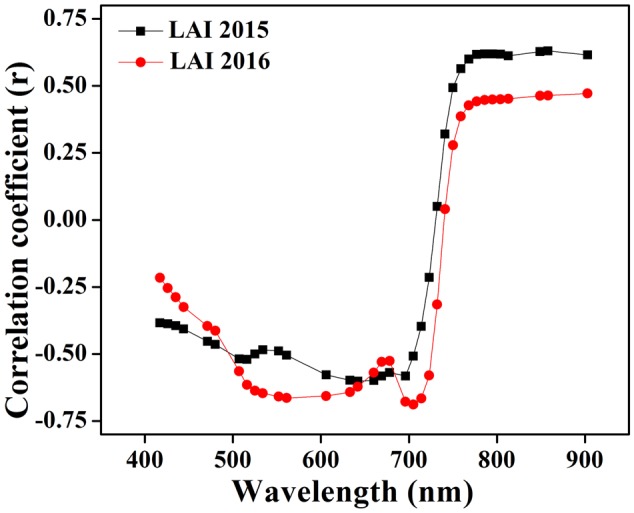
**Correlation among LAI and the canopy reflectance at wavelength from 440 to 900 nm using data collected in 2015 and 2016**.

The optimum combination of wavelengths used to calculate the VIs was derived from an analysis of the correlation between the LAI and canopy spectra. Strong relationships were shown (**Figure 2**) in the blue portion (440–475 nm), shorter green portion (500–550 nm), longer red portion (650–700 nm), and a particular portion of NIR (780–850 nm) (Darvishzadeh et al., 2009; Delegido et al., 2015). The same approach has already been applied at single phenological stage to estimate the chlorophyll, biomass as well as nitrogen contents with spectral reflectance in many crops of cotton, potato, soybean, and maize with coefficient of variation (CV) less than 25% and coefficient of determination (*R^2^*) higher than 0.80 (Nguy-Robertson et al., 2013). The bands included in the VIs are usually limited, and PCA has a better effect, which can make use of complementary advantages among different spectral bands (Ray et al., 2006).

Canopy spectral data have been used to developed several VIs to estimate biophysical parameters, e.g., LAI, chlorophyll and biomass (Delegido et al., 2013). Descriptions and formulas of VIs used in this study are listed in **Table 1**. RVI and normalized difference vegetation index (NDVI) have been frequently used to estimate LAI changes during the growth period due to simplicity, reduced influence of soil background and environmental noise (Pearson and Miller, 1972). However, the NDVI correlation with LAI can discriminate between the green leaf cellular structure, pigment and other canopy material (Rouse et al., 1973). GNDVI was found to be more sensitive to the leaf pigment concentration (Gitelson et al., 1996). BNDV1 has been developed to assess vegetation information but is seldom used because it is more easily affected by the atmosphere. However, atmospheric correction is applied to obtain accurate spectral reflectance (Wang et al., 2007).

**Table 1:** Descriptions and formulas of vegetation indices investigated in this study.

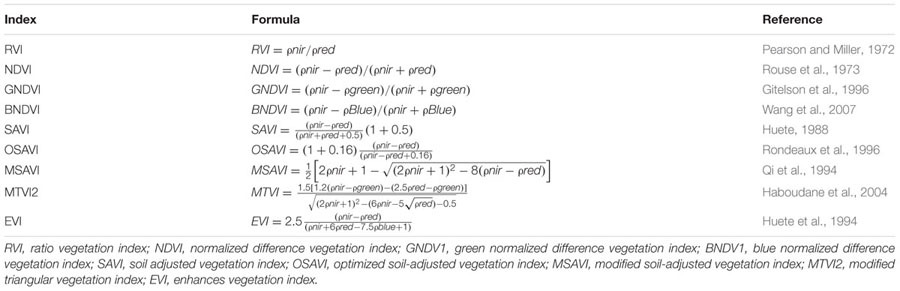

The vegetation canopy has been shown to be influenced by the soil background during phenological growth; therefore, SAVI was constructed to minimize the effect of the soil background (Huete, 1988). To enhance the sensitivity of the leaf pigment variability and reduce the soil background reflectance contribution, SAVI was improved into OSAVI (Rondeaux et al., 1996).

An improved form of SAVI with self-adjustment factor L was modified soil-adjusted vegetation index (MSAVI), which accounts for the difference in the soil background; however, this factor does not appear in the MSAVI formulation (Qi et al., 1994). The MSAVI construction based on the radiative transfer model is sensitive for canopy LAI estimation. Moreover, MSAVI has been proven to be less affected by a dense canopy variation and soil spectral properties. Modified triangular vegetation index (MTVI2) is an optimized form of MCARI and TVI that determines the function of green LAI. Furthermore, it preserves the LAI sensitivity as well as the resistance to chlorophyll reflectance (Delegido et al., 2013). To account for the sensitivity of the leaf biomass and minimize the effect of background sources, the enhances vegetation index (EVI) was constructed to optimize the vegetation signal with through a de-coupling of the canopy background signal and a reduction in atmosphere influences (Huete et al., 1994, 2002).

### Sensitivity Analysis

The determination coefficient (*R*^2^) and root mean square error (RMSE) were used to assess the predictive accuracy of the regression models (Govaerts et al., 1999). They constitute measurements of how good the regression models (best-fit function) are at capturing the relationship between LAI and VIs. When the best-fit function is non-linear, however, the *R*^2^ as well as the RMSE values may be misleading. The noise equivalent (NE) was used to determine the LAI estimation accuracy. The sensitivity of the VIs to the LAI was compared qualitatively using the following **(1)** expression of the NE. A NE with a diverse scale and dynamic limits proves its advantage compared to direct assessment among VIs and is thus used to verify the sensitivity of predicating LAI changes (Viña et al., 2011).

$$\begin{array}{c} \mathrm{NE}\Delta LAI=\frac{\mathrm{RMSE}\{\mathrm{VI}\mathrm{vs}.\mathrm{LAI}\}}{d(\mathrm{VI})/d(\mathrm{LAI})} \end{array}$$

where the RMSE is the RMSE of the best fit function between VI and LAI and d(VI)/d(LAI) is the first derivative in this relationship that is observed during the growing season (Gitelson, 2013). The NE method has already been applied to evaluate the sensitivity of the photosynthetic active radiation, chlorophyll contents, and vegetation fraction in wheat, maize, and soybean (Schlemmera et al., 2013).

## Results

### Variation of the Rice Canopy Reflectance Spectra under Different N Rates

**FIGURE 3:**
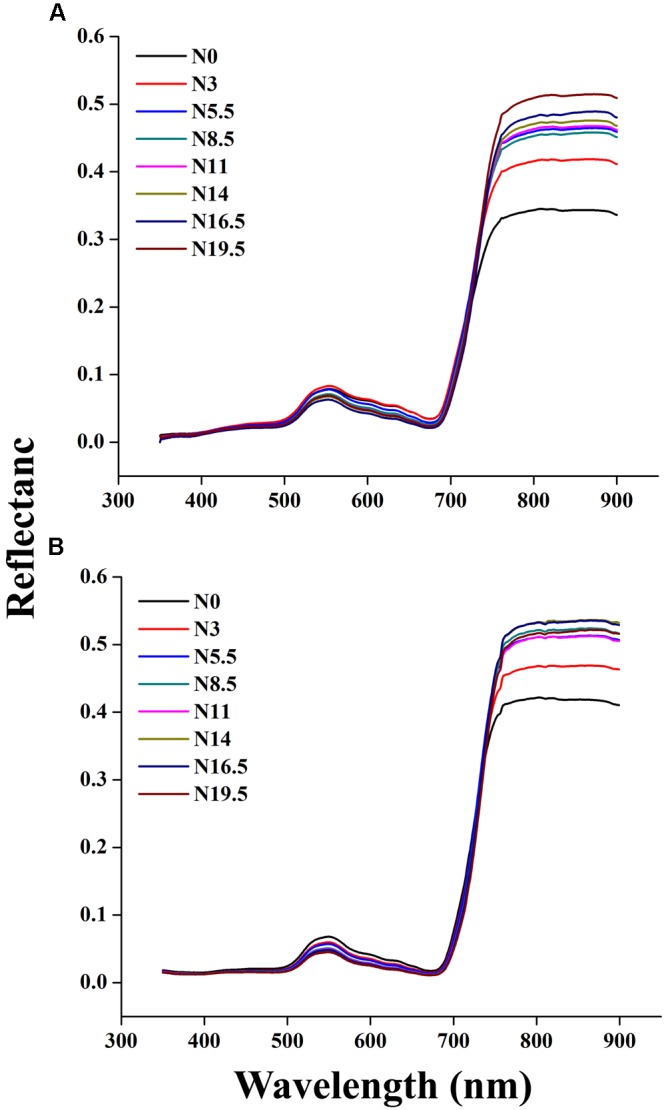
**Change in canopy reflectance spectra under different N rates at critical growth stages of rice in 2015 (A)** 2016 **(B)**, where N0, N3, N5.5, N8.5, N11, N14, N16.5, and N19.5 represents 0, 45, 83, 128, 165, 210, 248, and 293 kg N ha^-1^, respectively.

The canopy reflectance spectra of rice showed a marked variation under different N rates in each successive year, as shown in **Figures 3A,B**. The behavior of different regions of the spectrum changed due to the nitrogen response, analogous with other green plants. Our study demonstrated that nitrogen application from N 0 to N 19.5 over the crop phenological stages increased reflectance in the near infrared region (>720 nm) and reduced reflectance in the ultraviolet region (350–400 nm) as well as in the visible region (400–720 nm) of the spectrum. Within the visible region, the reflectance of the green region (490–560 nm) was always slightly higher than that of the violet-blue (400–425 nm) and the red region (640–685 nm). Moreover, the reflectance values near 680–900 and 530–560 nm result from the response of the rice canopy under different N rates. The impacts of increased leaf coverage and reduced soil and water below canopy on canopy reflectance were weakened by averaging all spectra measured under each N supply rate in whole growing season.

### Canopy Reflectance Spectra at Phenological Stages

**FIGURE 4:**
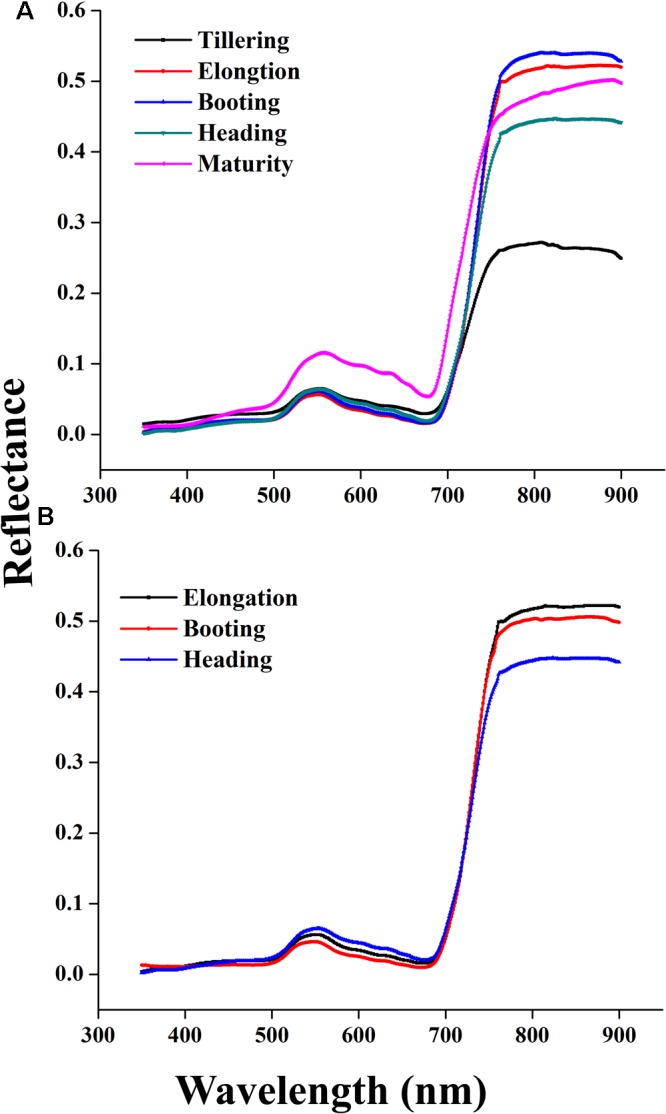
**Changes in canopy reflectance spectra from tillering to maturity of rice in 2015 (A)** elongation, booting, and heading stages in 2016 **(B)** under different N rates.

Canopy reflectance spectra were not constant but consistently changed at critical growth stages during the phenological period 2015 and 2016, as shown in **Figures 4A,B**. The variations of the canopy spectra revealed that from the elongation to heading stages of crop development, while the reflectance pattern showed a reducing trend in the visible region and increasing in the NIR region. In particular, the reflectance behavior of the red and green portion of the visible region changed due to the senescence of the crop. The reason for this change is that the dense canopy at early stages causes the increment in the green pigment, especially in leaves, to decrease reflectance. Thus, the crop phenological stages can be clearly identified. Leaf color variation, e.g., green-yellow when heading to maturity and nitrogen remobilization from leaf to grain at anthesis stage, caused reflectance increments in the NIR region under varying N strategies. Such variations of reflectance in the visible and near infrared regions were in agreement with the variations in the relationship between the coefficient of correlation and LAI over combined as well as individual critical growth stages of the rice crop as shown in **Figure 2**.

### Relationship of LAI to the Phenological Stages

**FIGURE 5:**
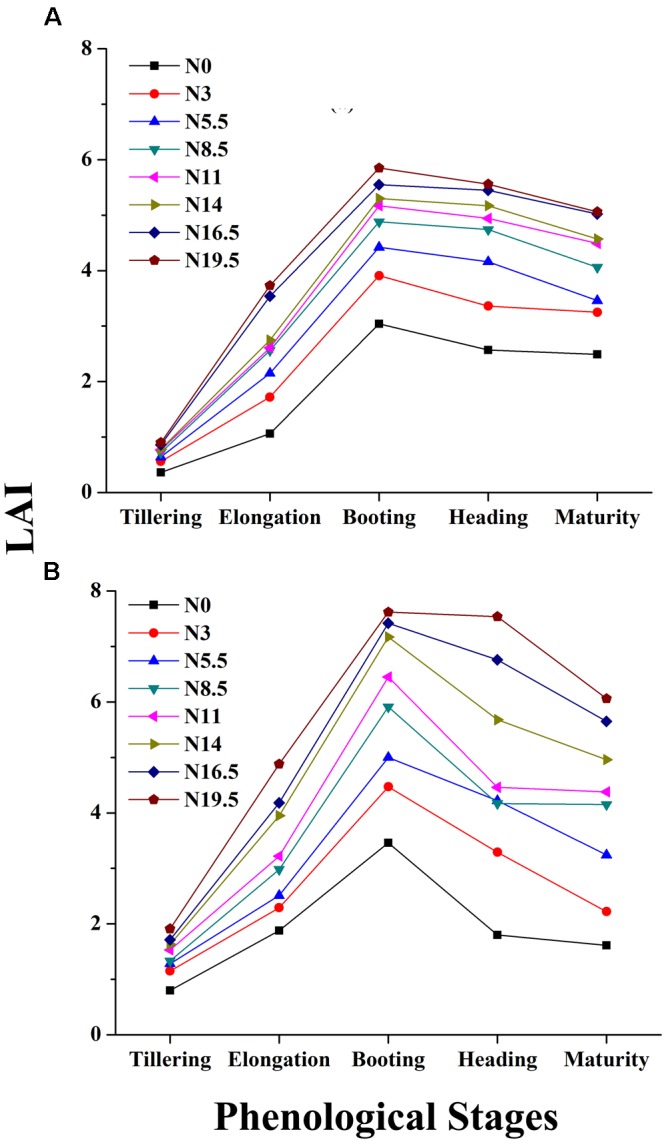
**Changes in LAI over the phenological period from tillering to maturity of rice under different N rates in 2015 (A)** and 2016 **(B)**, where N0, N3, N5.5, N8.5, N11, N14, N16.5 and N19.5 represents 0, 45, 83, 128, 165, 210, 248, and 293 kg N ha^-1^ respectively.

**FIGURE 6:**
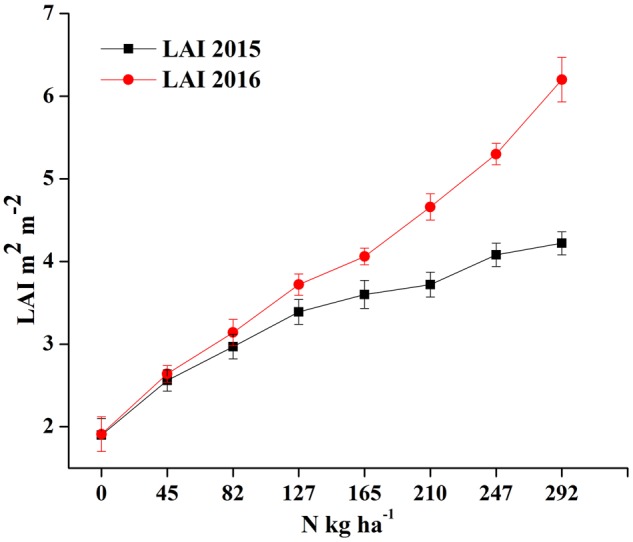
**Changes in LAI over two growing seasons in rice under varied N levels 0, 45, 83, 128, 165, 210, 248, and 293 kg N ha^-1^ in 2015 (A) and 2016 (B) respectively**.

The LAI measurements exhibit significant variation under different N rates, showing a consistent pattern among different phenological stages for the two crop years, as shown in **Figures 5A,B**. However, the LAI value over the growth period was higher in 2016 than in 2015 under the same N rates, presumably due to the relatively higher soil fertility status in the later growing season, as shown in **Figure 6**.

Leaf area index increases with zero-N to higher-N rates as plants develop from tillering to maturity. The leaf appearance was more erect in plots with zero-N rates characterized by lower LAI, and all of the changes were manifested in the canopy reflectance. The LAI reaches its peak before heading and goes down with plant senescence, demonstrating significant leaf area changes related to the phenological stages. The increased LAI before heading was due to the expansion of the leaf photosynthetic area, building of carbohydrates, which improve the leaf area, leaf dry weight and overall plant biomass.

The ranges of the LAI in the entire dataset were (0.19–7.62 m^2^ m^-2^) in 2015 and 2016 (**Table 2**). In 2015, the LAI gradually increased along with plant growth from an average value of 0.36 m^2^ m^-2^ at tillering with zero-N to 5.06 m^2^ m^-2^ at maturity with maximum N-application. The significant LAI difference between the zero-N treatment and high-N application (292 kg ha^-1^) occurred at booting, with a value of 5.85 m^2^ m^-2^ in 2015. This trend of variation was also true for 2016 across all phenological stages (**Table 2**). Across the phenological stages, the LAI value ranged from 0.80 to 6.78 m^2^ m^-2^ with 0–292 N kg ha^-1^, and the maximum 7.62 m^2^ m^-2^ was recorded at booting stages under higher (292 N kg ha^-1^) N application. The variation range of LAI demonstrated a significant change in the absorption of the red and green regions and reflectance in the NIR region due to changes in the leaf area over the growth period. Moreover, dynamic changes in the leaf area and its contents, such as chlorophyll, over the growth period could also be a good indicator of non-destructive N measurement and management. The LAI ranges in both years (**Table 3**) were sufficiently broad to evaluate the potential ability of the VIs; however, the dataset shows that the LAIs had a specific range of values at each phenological stage.

**Table 2:** Description of *Oryza sativa* L. leaf area index (LAI) at phenological stages under different nitrogen rates.

	LAI m^2^ m^-2^ 2015					LAI m^2^ m^-2^ 2016				
N rates	Tillering	Elongation	Booting	Heading	Maturity	Tillering	Elongation	Booting	Heading	Maturity
0 N kg ha^-1^	0.36 f	1.06 h	3.04 h	2.57 h	2.49 h	0.80 e	1.88 e	3.46 f	1.81 g	1.60 e
45 N kg ha^-1^	0.56 e	0.56 g	3.91 g	3.36 g	3.25 g	1.15 d	2.29 de	4.47 e	3.07 f	2.22 e
82 N kg ha**^-^**^1^	0.64 d	2.15 f	4.42 f	4.16 f	3.46 f	1.28 cd	2.51 d	4.90 e	3.69 e	3.24 d
127 N kg ha**^-^**^1^	0.72 c	2.56 e	4.88 e	4.74 e	4.01 e	1.33 cd	2.98 c	5.91 d	4.22 de	4.15 c
165 N kg ha**^-^**^1^	0.77 b	2.61 d	5.17 d	4.94 d	4.49 d	1.53 bc	3.22 c	6.45 cd	4.65 d	4.46 c
210 N kg ha**^-^**^1^	0.78 b	2.75 c	5.30 c	5.17 c	4.57 c	1.63 b	3.95 b	7.17 c	5.68 c	4.90 bc
247 N kg ha**^-^**^1^	0.86 a	3.54 b	5.55 b	5.45 b	5.02 b	1.71 ab	4.18 b	7.42 b	6.76 b	5.42 b
292 N kg ha**^-^**^1^	0.90 a	3.73 a	5.85 a	5.56 a	5.06 a	1.91 a	4.88 a	7.62 a	7.84 a	6.78 a

*In a column, means followed by the letters a–h denote significant difference at 5% level by ANOVA.*

**Table 3:** Summery statistics of leaf area index (LAI) at phenological stages under different nitrogen rates in 2015 and 2016.

	LAI m^2^ m^-2^ 2015			LAI m^2^ m^-2^ 2016		
N rates	*Mean ± SD*^†^	Minimum	Maximum	*Mean ± SD*	Minimum	Maximum
0 N kg ha^-1^	1.90 + 0.15	0.36	3.04	1.62 + 0.52	0.19	3.46
45 N kg ha^-1^	2.56 + 0.23	0.56	3.91	2.27 + 0.25	0.24	4.47
82 N kg ha^-1^	2.97 + 0.22	0.64	4.42	2.76 + 0.45	0.32	5.00
127 N kg ha^-1^	3.39 + 0.39	0.72	4.88	3.15 + 0.33	0.34	5.91
165 N kg ha^-1^	3.60 + 0.21	0.77	5.17	3.40 + 0.21	0.38	6.45
210 N kg ha^-1^	3.72 + 0.30	0.78	5.30	3.96 + 0.37	0.37	7.17
247 N kg ha^-1^	4.08 + 0.37	0.86	5.55	4.54 + 0.30	0.51	7.42
292 N kg ha^-1^	4.22 + 0.27	0.90	5.85	5.16 + 0.42	0.64	7.62

*^†^Mean ± SD, mean and standard deviation.*

### Relationship of Vegetation Indices to the Phenological Stages

The spectral indices expressed the variation on the canopy development from elongation to heading, temporal signature of RVI (14.93–9.48) and NDVI (0.86–0.80) changes in 2015 and same pattern of variation (12.85–8.88) in RVI and NDVI (0.82–0.81) observed in 2016 from elongation to heading probably due to initiation of panicle in canopy, Moreover, the NDVI and RVI tend to saturate easily with the progress of canopy growth (**Table 4**). SAVI and OSAVI expressed better but similar behavior less sensitive to canopy biophysical parameters (0.73–0.66) from booting to heading in 2015 and (0.75–0.63) in 2016 at same growth stages as compared to MSAVI and MTVI which are modified index, and values comprised 0.73–0.62 and 0.074–0.63 from elongation to heading and in 2015 and 2016. GNDVI value ranged 0.84–0.80 from elongation to heading in 2015 and 0.87–0.82 in 2016 at same phenological stage. While the BNDVI showed relatively higher values of from elongation to heading in each year. This variation vegetation of indices might be due to leaf angle within the canopy rather than the individual leaf reflectance properties at different phenological stage.

**Table 4:** Relationship of vegetation indices to the phenological stages in 2015 and 2016.

	Phenological stages					
	2015			2016		
Vegetation indices	Elongation	Booting	Heading	Elongation	Booting	Heading
RVI	14.93 ± 4.53^†^	13.92 ± 3.20	9.48 ± 1.86	12.85 ± 4.08	13.82 ± 0.59	8.88 ± 0.49
NDVI	0.86 ± 0.04	0.86 ± 0.04	0.80 ± 0.04	0.82 ± 0.47	0.84 ± 0.58	0.81 ± 0.70
GNDVI	0.84 ± 0.04	0.84 ± 0.04	0.80 ± 0.03	0.87 ± 0.52	0.84 ± 0.61	0.82 ± 0.57
BNDVI	0.93 ± 0.02	0.93 ± 0.02	0.92 ± 0.01	0.93 ± 0.47	0.90 ± 0.27	0.88 ± 0.24
SAVI	0.73 ± 0.06	0.74 ± 0.06	0.66 ± 0.05	0.74 ± 0.48	0.73 ± 0.38	0.68 ± 0.30
OSAVI	0.86 ± 0.04	0.87 ± 0.04	0.82 ± 0.03	0.87 ± 0.47	0.86 ± 0.37	0.83 ± 0.31
MSAVI	0.73 ± 0.09	0.74 ± 0.08	0.62 ± 0.07	0.79 ± 0.54	0.74 ± 0.48	0.69 ± 0.42
MTVI2	0.71 ± 0.09	0.71 ± 0.09	0.58 ± 0.07	0.74 ± 0.53	0.73 ± 0.50	0.63 ± 0.50
EVI	0.74 ± 0.09	0.75 ± 0.09	0.61 ± 0.07	0.75 ± 0.58	0.76 ± 0.49	0.63 ± 0.45

*^†^Mean ± SD, mean and standard deviation.*

### Evaluation of Vegetation Indices for Estimation of Rice LAI at Phenological Stages

**FIGURE 7:**
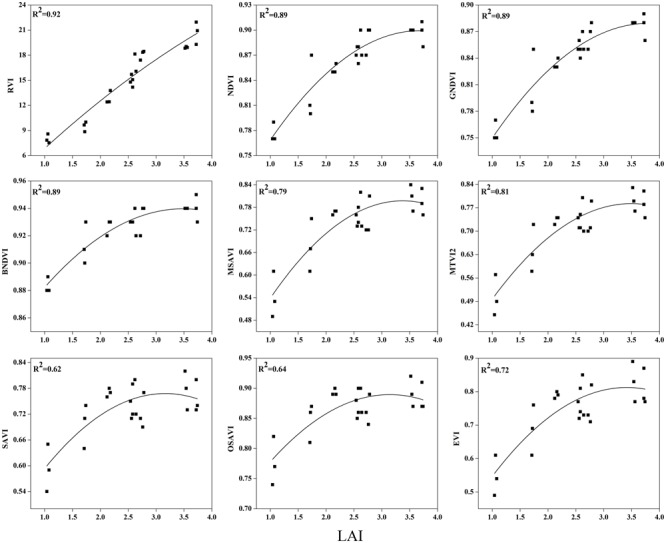
**Best-fit models between vegetation indices (VI) and LAI at elongation stage**.

**FIGURE 8:**
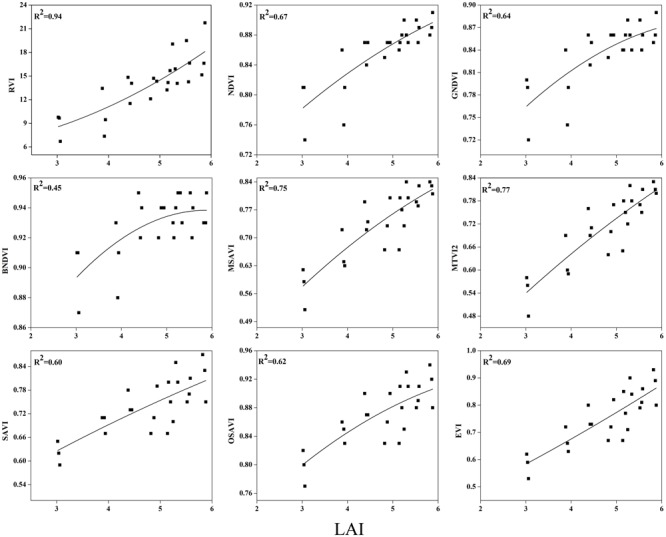
**Best-fit models between VIs and LAI at booting stage**.

**FIGURE 9:**
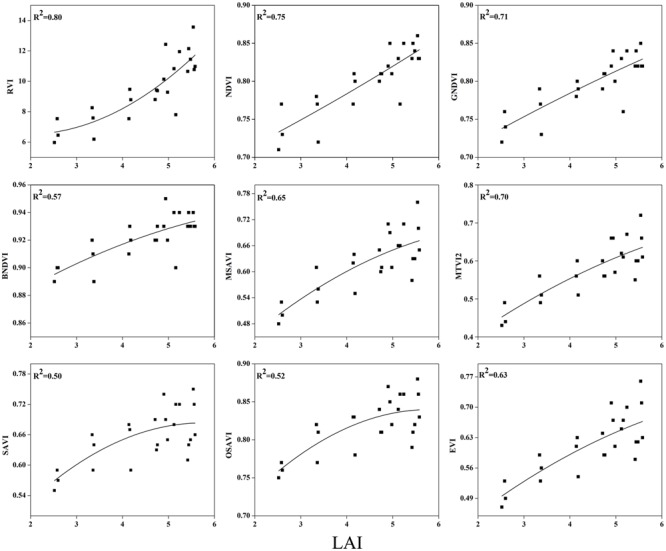
**Best-fit models between VIs and LAI at heading stage**.

To examine the suitability of the canopy reflection feature for assessing the LAI, the corresponding best fit models of the relationships between all of the VIs and LAI were discriminated at each phenological stage shown in **Figures 7**–**9**. Typical patterns of change in different phenological stages demonstrated the potential utility of these indices. The first part in **Table 5** shows the *R^2^* of the estimation equation for the relationship between the LAI and VIs for 2015 and 2016. The results indicated that all of the indices were increasingly related to the temporal distribution of the LAI data of 2015 and 2016 at different phenological stages, except for tillering and maturity, which showed the lowest *R^2^* values for all of the indices and were excluded from the data. Among the nine indices, the RVI captured the LAI, with the highest determination coefficient (*R^2^* = 0.92), followed by NDVI, BNDVI, and GNDVI (*R^2^* = 0.89) at the elongation stage. By contrast, SAVI and OSAVI exhibited (*R^2^* = 0.62, 0.64) less significant relationships with LAI at the elongation stage as shown in **Table 5**. The RVI (*R^2^* = 0.94), followed by MTVI2, MSAVI, EVI, and NDVI (*R^2^* = 0. 77, 0.75, 0.69, and 0.67, respectively), had the best relationship, but the least was observed with BNDVI (*R^2^* = 0.45) at the booting stages. The next heading phenological stage provides best-fit models of the LAI with the VIs RVI (*R^2^* = 0.80), NDVI (*R^2^* = 0.75), and GNDVI (*R^2^* = 0.71), followed by MTVI2 (*R^2^* = 0.70) and MSAVI (*R^2^* = 0.65). However, when the LAI exceeded saturation (LAI > 3 m^2^ m^-2^) at the booting stage, the NDVI leveled off (*R^2^* = 0.67), and its sensitivity suffered while the MSAVI (*R^2^* = 0.75) and MTVI2 (*R^2^* = 0.77) have modifying factor to cope with it. All of the indices approached high values at higher doses of N from tillering to booting and diminished from heading to maturity.

**Table 5:** Validation of *R^2^* and RMSE for estimation of LAI at phenological stages.

	*R^2^* val 2015			*R^2^* val 2016			RMSE		
Vegetation indices	Elongation	Booting	Heading	Elongation	Booting	Heading	Elongation	Booting	Heading
RVI	0.92	0.94	0.80	0.72	0.49	0.39	0.32	0.28	0.24
NDVI	0.89	0.67	0.75	0.77	0.69	0.48	0.30	0.28	0.21
GNDVI	0.89	0.64	0.71	0.72	0.46	0.45	0.40	0.29	0.34
BNDVI	0.89	0.45	0.57	0.57	0.25	0.34	0.42	0.30	0.36
SAVI	0.62	0.60	0.50	0.42	0.35	0.31	0.36	0.37	0.34
OSAVI	0.64	0.62	0.52	0.42	0.34	0.33	0.35	0.36	0.24
MSAVI	0.79	0.75	0.65	0.56	0.55	0.46	0.30	0.31	0.22
MTVI2	0.81	0.77	0.70	0.59	0.58	0.49	0.32	0.31	0.23
EVI	0.72	0.69	0.63	0.52	0.47	0.46	0.34	0.31	0.23

*RVI, ratio vegetation index; NDVI, normalized difference vegetation index; GNDV1, green normalized difference vegetation index; BNDV1, blue normalized difference vegetation index; SAVI, soil adjusted vegetation index; OSAVI, optimized soil-adjusted vegetation index; MSAVI, modified soil-adjusted vegetation index; MTVI2, modified triangular vegetation index; EVI, enhances vegetation index.*

### Validation of Estimation between the Vegetation Indices and LAI

**FIGURE 10:**
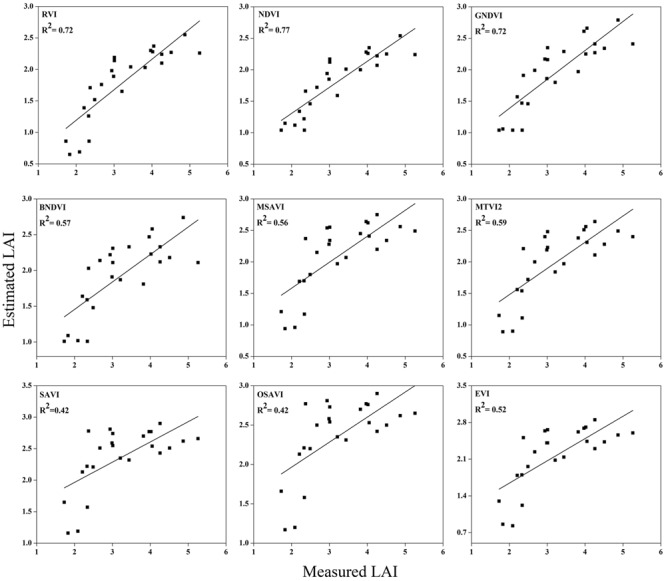
**Relationship between measured LAI and estimated LAI at elongation**.

**FIGURE 11:**
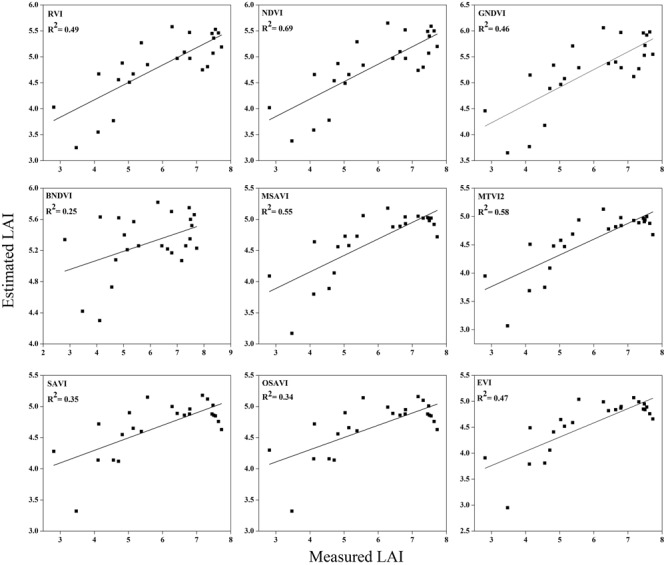
**Relationship between measured LAI and estimated LAI at booting**.

**FIGURE 12:**
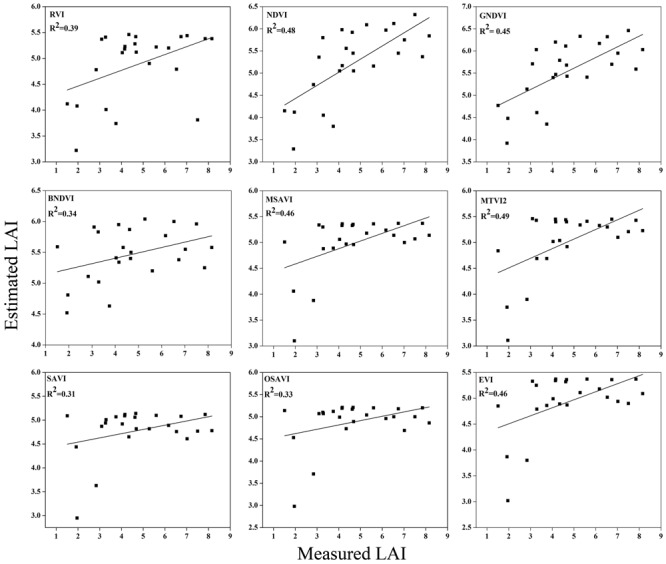
**Relationship between measured LAI and estimated LAI at heading**.

To test the above calibration models, relationships between the VIs and LAI, potential validation with the subsequent year 2016 data (*n* = 120) was applied, showing the highest values at different phenological stages (**Figures 10**–**12**). The regression equation derived from the calibration data from 2015 were applied to validate the 2016 data and resulted in the estimation of the paddy LAI. The *R^2^* of the calibration and *R^2^* of the validation at three phenological stages were calculated for all of the models for the datasets from both years. The predictive ability of VIs to assess the LAI was identified by RMSE and a sensitivity analysis. The *R^2^* and RMSE results were better and fluctuated with the phenological stages, as summarized in **Table 5**. Moreover, it is common that the results of the estimation of the LAI with calibration data are better than those of the validation data.

The equations with the first two maximum *R^2^* were fitted between the LAI and RVI (0.72) and NDVI (0.77) or GNDVI (0.72) have strong correlations, and the relationship between SAVI, OSAVI, and EVI exhibited a smaller correlation (0.42, 0.42, and 0.52) at the elongation of crop development. At the booting stage NDVI (0.69) and MTVI2 (0.58) shows best fit with LAI followed by MSAVI (0.55) and RVI (0.49). VIs MTVI2 (0.49) and NDVI (0.48) followed by MSAVI (0.46) are strongly correlated to the LAI at heading stage. The uppermost values were ≤0.44 for the RMSE for all of the indices. The BNDVI, GNDVI, and SAVI (0.42, 0.40, and 0.36) exhibited the highest RMSE at the elongation of the vegetative phase. At booting NDVI, RVI both have least RMSE (0.28). The results indicate that all indices are closely related to the LAI and thus have a high potential to provide an accurate LAI estimation over a temporal distribution. Therefore, it is not necessary that the best-performing indices at each stage perform best at each phenological stage and that all of the indices behave uniformly at all stages performed best with LAI estimation. The best fit least RMSE was observed for the NDVI (0.21), MTVI2 (0.23), and MSAVI (0.22) at heading stages of crop development. The performance of the VIs fluctuated with the growth stage, but most of the indices had the best performance at later growth stages when the canopy was partially closed, as at earlier stages, the field was fully covered by the canopy. VIs constructed from green, blue, and red band combination reflectance show a better relationship with the LAI at early stages.

In addition, the results of our field study suggest that an analysis of the field LAI with the canopy spectral reflectance at three phenological stages, e.g., elongation, booting, and heading, might be used to evaluate the potential of LAI-related VIs.

### Sensitivity Analysis

**FIGURE 13:**
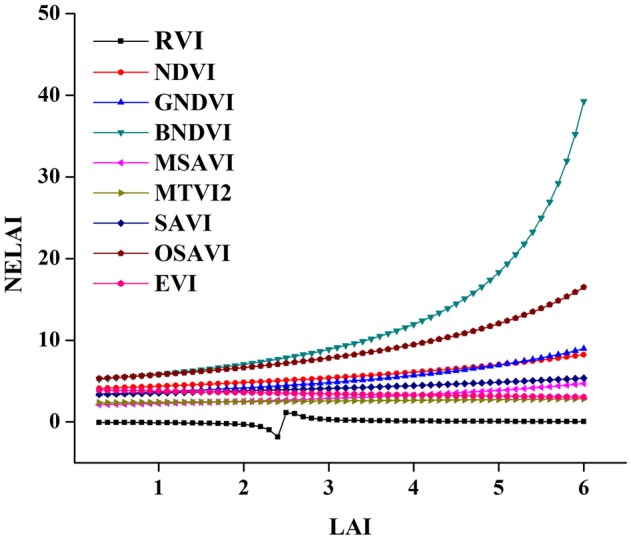
**Sensitivity analysis for nine spectral VIs tested to LAI**.

In this study, we evaluate the potential relationship of eight VIs vs. LAI over the phenological periods of a rice crop and test the sensitivity to overcome the decreased sensitivity of VIs at varying N levels (**Figure 13**). BNDVI and OSAVI exhibited the highest insensitivity. EVI and RVI showed moderate insensitivity, while MTVI2 was consistent. A significant decrease in the sensitivity of NDVI was observed when the LAI exceeded three concealing changes in vegetation with moderate to high levels (Yang et al., 2010; Nguy-Robertson et al., 2014). As the LAI > 3 NE of both NDVI and GNDVI, however, decreased the NE of NDVI, which remained below that of GNDVI. NEs for MSAVI and MTVI2 were similar and were linearly related to the LAI (Dong et al., 2016). The sensitivity of SAVI and OSAVI are soil-related indices and are relatively inconsistent over the entire range of the LAI, so they are not included in the representation. EVI and RVI are better indices for numerical changes in the LAI as the other indices are frail at higher values of LAI. On the basis of the high sensitivity and degree of linearity, MSAVI and MTVI2 might be useful for LAI estimation.

## Discussion

The results were supported by the hypothesis that multiple peak regions of the canopy spectral reflectance formulate many narrow band VIs, which have the potential to be used to evaluate crop biophysical parameters. The reflectance around the peaks was used to extract accurate information to improve the quantitative accuracy of the VIs used to evaluate crop attributes, such as LAI, N status, biomass, and so on Li et al. (2013). Such regions of the canopy spectra have been observed in many crops, such as wheat, maize, soybean, cotton, and grass (Perry et al., 2012). Up to five vegetation stages have already been identified in both maize and soybean using spectral reflectance under varying rates of fertilizers (Morier et al., 2015). In the current study, the potential of using nine VIs with the rice LAI was investigated at different phenological stags under varying N fertilization levels and showed that the progress of the LAI from tillering to heading has a reasonable association with the canopy reflectance characteristics in rice (**Figures 4, 5**), which has been reported previously for cultivation (Maki and Homma, 2014; Xiong et al., 2016).

Canopy reflectance reaches its peak value before heading and decreases later, at senescence. Transformation in the canopy reflectivity of different spectral parts is concurrent with the LAI and is often confounded through modification of the leaf chlorophyll contents during plant growth with increasing N fertilization (Jégo et al., 2012). Elevated N application generates taller plants with higher numbers of tillers and a greater leaf area, which requires more carbohydrates in the plant canopy (Pan et al., 2011). Not only the biophysical characteristics of vegetation, canopy architecture, atmospheric absorption, and scattering affect the canopy hyperspectral reflectance but so do the direction of incidence radiation and soil backgrounds (Carvalho et al., 2016). During early phenological periods of our rice crop, variations in the maximum spectral reflectance in the visible region are likely to be small, which might be due to the soil water background and nitrogen contents in the canopy under varying N application. However, predominant changes in the NIR reflectance with changes in the leaf orientation from horizontal to vertical at certain stages of the growth cycle in rice owing to overlapping leaves reduced the active photosynthetic size after the increment of the LAI reached a plateau, in accordance with previous studies (Tian et al., 2009, 2013; Huang et al., 2013). Younger rice plants absorb more photosynthetically active radiation from the visible region due to the formation of new tissue (until all tillers have reached the flag leaf emergence stage) and chlorophyll filling, and they reflect more NIR due to chlorotic and necrotic turning during senescence, as has been previously reported (Feng et al., 2014; Gaju et al., 2014). Moreover, the visible regions of the spectral spaces are useful for estimating the vegetation fraction in multi-crop and background datasets (Gitelson et al., 2002). The LAI was maximum at the booting stage after the subsequent loss in the LAI with each growth step (Liu et al., 2012) due to the loss in photosynthetic activity under the same environmental conditions (Wu et al., 2016). The rice yield was positively correlated with the LAI near the heading, maintaining the larger leaf area duration before heading until harvesting (Song et al., 2011).

High values of LAI in successive years at phenological stages corresponding to previous studies might be due to better inherent N fertility, soil N balance and crop background. Previously, hyperspectral reflectance screened growth changes of wheat crop in response to NPK applications (Mahajan et al., 2014).

Vegetation indices, such as NDVI and RVI, demonstrated close relationship with the LAI as the LAI was at approximately 3 m^2^ m^-2^ (Zhang et al., 2015). Reduced variability in red reflectance *p*_red_ and NIR reflectance *p*_NIR_ when the LAI approaches the saturation point and formulation of the NDVI has *p*_red_, and *p*_NIR_ makes it insensitive. While GNDVI could not obviously reach a saturation level, even the LAI value was approximately moderate to high (4–5.5). This result provides more accurate information for assessing the LAI under different nutritional statuses as well as different phenological stages of paddy crop. Moreover, accurate estimation of the LAI could be completed with blue and green bands compared to red, when even the LAI should be greater than 3 m^2^ m^-2^ at the elongation stage (Li et al., 2010; Motohka et al., 2010; Inoue et al., 2012), suggesting that the blue region could improve the ability to estimate the LAI across different phenological stages in rice and wheat. However, the maximum NIR reflectance region response cannot be neglected under changing rice growing conditions (Hatfield and Prueger, 2010). The NIR band has a strong contribution to strengthening relationships between the spectral reflectance and LAI (Darvishzadeh et al., 2009). At the late reproductive phase, reflectance in the NIIR region represents a supplementary increase over the red region due to additional vegetative growth of spikes, resulting in an increase in the NDVI value at this stage (Gitelson et al., 2014). The notable raise in NDVI is evident in our study during the reproductive phase, where additional vegetative growth or the development of a spike during later growth could contribute to the reflectance. These findings demonstrated once again that the spectral reflectance and VIs were sensitive to early and late reproductive growth due to the senescence effect (Gitelson et al., 2005; Li et al., 2016). The NE was applied as an accuracy indicator and was used to verify the performance of vegetative indices for LAI estimations in the field; moreover, it accounts for both a scattering point from the slope and the slope of the best fit function (Saltelli et al., 2010; Xiao et al., 2014). Non-linearity of the best-fit function between the VIs and LAI at the phenological periods shows the irregular pattern of sensitivity. The NE is a better index for assessing the sensitivity of spectral parameters and the LAI because the RMSE and *R^2^* values can be misleading about the estimation accuracy of the LAI (Heiskanen et al., 2013; Marshall et al., 2016).

## Conclusion

The LAI a more sensitive growth parameter for spectral reflectance and VIs at vegetative than in reproductive stage due to senescence effects. Our results demonstrate that for rice leaf characterization reflectance at various band such as infrared (>760 nm) and visible (524–534, 583, 687, and 707 nm) are most important. At the vegetative phase of the crop, elongation or stand establishment, the LAI shows the maximum relationship with the spectral reflectance and VIs among phenological stages with eight nitrogen fertilization levels. The RVI and NDVI exhibited significant potential for LAI estimation, followed by MSAVI and MTVI2 at the most critical elongation, the booting and heading growth stages. RVI and EVI showed changes in LAI sensitivity that was capable of detecting differences in treatments during the senescence phase, which caused a more rapid loss of leaf area. Moreover, potential evaluation of VI through the sensitivity analysis technique is not only suitable for the investigation of VIs but can also be extended to other climatic, ecological, and environmental variables. Using the changes in the LAI and spectral sensitivity at phenological stages, three stages were identified to evaluate the potential of VIs. Future studies should be directed toward determining other species and crops as well as to changing phenological growth and other biophysical parameters under varying N rates.

## Author Contributions

MD and SW originally formulated the idea and designed the experiments. MD and WZ performed the field experiment and hyperspectral measurements and did the laboratory analyses. MD and SW performed the statistical analyses and wrote the manuscript. MR and ZS provided editorial support and advice.

## Conflict of Interest Statement

The authors declare that the research was conducted in the absence of any commercial or financial relationships that could be construed as a potential conflict of interest.
